# Leaving the labor market: Exit routes, personality traits and well-being

**DOI:** 10.1371/journal.pone.0263670

**Published:** 2022-03-30

**Authors:** Dusanee Kesavayuth, Robert E. Rosenman, Vasileios Zikos

**Affiliations:** 1 Department of Economics, Faculty of Economics, Kasetsart University, Bangkok, Thailand; 2 School of Economic Sciences, Washington State University, Pullman, WA, United States of America; 3 Faculty of Economics, Chulalongkorn University, Pathumwan, Bangkok, Thailand; University of Luxembourg and Luxembourg Institute of Socio-Economic Research (LISER), LUXEMBOURG

## Abstract

In this paper we examine the buffering effects of personality traits when people leave their work in later life. Using large-scale panel data for the UK, we show that depending on the exit route and satisfaction related to overall life and the domains of income and leisure, different personality traits act as moderators. Besides augmenting leisure satisfaction for those who hit mandatory retirement, conscientiousness augments life satisfaction for those becoming unemployed. On the contrary, extraversion mitigates satisfaction with life, income, and leisure for those who retire early. This may be an indication that extraverted individuals who tend to be sociable and outgoing may suffer when losing social relationships from their work. At the same time, extraversion may be helpful in augmenting leisure satisfaction for those who stop working for reasons related to ill health or family care. Neuroticism augments income satisfaction for those who become unemployed, which may reflect that people high in neuroticism had a lower “baseline level” of income satisfaction relative to typical individuals so they were not affected as much. Finally, agreeableness mitigates life and leisure satisfaction for those hitting mandatory retirement, as is also the case with openness in terms of income satisfaction.

## 1. Introduction

From 1980 to 2017 the world’s population of those 60 years and over went from 382 million to 962 million [1]. Projections show that the proportion of older people is likely to double again by 2050. As a result of this demographic change, there is increased interest in older people’s health and well-being as they transition out of the workforce. Some studies document that retirement has a positive impact on individual well-being [2–4], but others find only little effect [5, 6]. Bonsang and Klein [7] make a distinction between voluntary and involuntary retirement, showing that although the former has a negligible effect on life satisfaction, the latter implies a strong and negative impact. However, the main focus of these studies is on retirement and therefore they do not distinguish between different exit routes from the labor market.

Traditionally retirement was perceived as the transition from being a full-time employee to becoming recipient of a pension. This still appears to be the case for most Europeans between the ages of 50–69. But the exit pathways are different for some people who leave the labor market to fulfill domestic tasks, due to ill health, or because they become unemployed. For example, among older Europeans leaving the labor market in 2012, 8% exited because they had lost their job while over 22% stopped working for health-related reasons including disability, a share exceeding 30% in some countries like Portugal, Estonia and Norway [8, 9].

A recent and growing empirical literature explores explicitly how different pathways out of employment affect different domains of life satisfaction. For example, Hetschko et al. [10] show that involuntary unemployment between a person’s last job and retirement leads to reduced well-being after retirement, which cannot be explained by changes in income. Involuntary unemployment thus leaves a scar on well-being that may persist after retirement. However, this does not appear to be the case for those who influenced or initiated unemployment as they did not show any scarring. In another related study, Palomäki [11] uses data from Europe over the period 2010–2013, and shows that the specific retirement route may have significant implications for individuals’ financial satisfaction even after controlling for income. Wetzel et al. [12] find that transitioning into retirement is accompanied by a short-term increase in life satisfaction, an effect that is more pronounced among those who were previously unemployed. In contrast, Hyde et al. [13] and Halleröd et al. [14] show that the retirement pathway in itself has little effect on post-retirement health and well-being. Given these conflicting results, there is a need for further research to better understand the link between exit pathways and individual well-being.

Economists and other social scientists are becoming increasingly interested in the study of individuals’ personality traits [15–20]. For example, Almlund et al. [17] compare personality to cognitive abilities for predicting a battery of social and economic outcomes, interpreting personality as a construct derived from an economic model of preferences, constraints and information. After controlling for family background and cognition, the authors conclude that personality measures are just as predictive as cognitive measures for many outcomes, and in fact influence standard measures of cognitive achievement.

There is more and more empirical evidence about the importance of personality traits for social and economic outcomes (see [15] for wages; [21] for job search behavior; [22] for unemployment duration; and [23] for well-being). A consistent finding is that personality affects how people react to discrete life events like unemployment [24], income changes [25], marriage, childbirth and widowhood [26], illness [27] as well as other events that may take place in our lives [28].

But only a handful of studies assess the buffering effects of personality when people leave their work in later life. Robinson et al. [29] use an on-line survey design with 365 participants who were observed at one time-point, and show that agreeableness, conscientiousness and low neuroticism are the most significant predictors of life satisfaction among retirees. Kesavayuth et al. [30] find that the life satisfaction of retired females high in openness or low in conscientiousness is higher compared to other females. However, these studies focus only on retirement and therefore provide no guidance on how other exit pathways from the labor market might affect people’s well-being. Considering different pathways, and how they interact with personality to determine satisfaction with overall life and the domains of income and leisure, provides finer distinctions that allow us to see if individuals react differently to the experience of leaving their work in later life. Such distinctions in turn might be particularly useful for policy makers in aiding the design or targeting of interventions that promote people’s well-being.

The current study draws large-scale panel data from the British Household Panel Survey (BHPS). To preface our results, we show that besides augmenting leisure satisfaction for those who hit mandatory retirement, conscientiousness augments life satisfaction for those becoming unemployed. By contrast, extraversion mitigates satisfaction with life, income, and leisure for those who retire early. At the same time, extraversion may be helpful in augmenting leisure satisfaction for those who stop working for reasons related to ill health or family care. Neuroticism augments income satisfaction for those who become unemployed. Agreeableness mitigates life and leisure satisfaction for those hitting mandatory retirement, as is also the case with openness in terms of income satisfaction. These findings provide a new perspective regarding the importance of personality on satisfaction with overall life and the domains of income and leisure when older individuals exit the labor market through alternative pathways.

## 2. Background and expectations

Leaving the labor market at an older age is a major life course transition that is often accompanied by changes in different areas of a person’s life. To examine the role of different exit pathways, and how they interact with personality to determine satisfaction with overall life and the domains of income and leisure, one first needs to develop a conceptual understanding of these relationships.

For this purpose it is useful to consider the classical life-cycle model (see e.g., [7, 31, 32]). This model posits that utility is a function of both consumption expenditure and labor supply. Individuals seek to maximize the discounted sum of their utilities through the choice of these two variables. The life-cycle model assumes that consumption is smoothed over time: income exceeds consumption before leaving the workforce at an older age, and consumption exceeds income thereafter. Furthermore, the exit from the workforce can be either voluntary or involuntary, the latter reflecting an unexpected shock including unemployment and sickness. In this paper, we consider four pathways of leaving work: early retirement, mandatory retirement, ill health or family care, and unemployment. While early retirement is voluntary, mandatory retirement is involuntary as, by definition, means no choice. Likewise, ill health/family care and unemployment are typically not a choice for most people and thus can be considered as involuntary as well.

According to the life-cycle model, a voluntary exit from the workforce brings about a decline in both consumption and income while the amount of leisure time increases [7]. In a similar vein, when individuals leave the workforce involuntarily, both consumption and income decrease, and the amount of leisure time increases. However, the drop in both consumption and income after leaving the labor force is larger compared to the case that the exit is voluntary.

Considering these effects in terms of satisfaction, a voluntary exit from the labor force is expected to reduce income satisfaction while increasing satisfaction with the amount of leisure time. An involuntary exit from the labor force, similar to a voluntary exit, is also expected to reduce income satisfaction while increasing leisure satisfaction.

In this context, one can consider the role of personality traits as a psychological buffer. To do this, and formulate specific hypothesis, it is useful to look at the definition of each trait within the taxonomy of the Big Five factor model (see [33], for a review). According to this model, conscientiousness describes the attribute of having self-control, being organized, responsive and proactive. Agreeableness reflects the quality of interpersonal relationships, and individuals high in agreeableness tend to act in a cooperative manner. Extraversion relates to the quantity and intensity of relationships; extraverted individuals tend to be sociable, outgoing, talkative and excitement-seeking. Openness is characterized by creativity and openness to new intellectual, cultural or aesthetic experiences. Last, Neuroticism is associated with characteristics like rapid mood changes and negative emotional reactions.

Based on these definitions, we expect that all personality traits but neuroticism would augment the positive effects associated with the different pathways of leaving the workforce while mitigating the negative ones. In other words, we believe that individuals high in agreeableness, conscientiousness, extraversion, and openness but low in neuroticism may find it easier than typical individuals to cope when they stop working. At the same time, there is a possibility that agreeableness and extraversion may not be helpful. Individuals scoring high on those traits may suffer psychologically from losing social relationships from their work. In a similar vein, openness may make it more difficult to cope; individuals with high openness levels who value exposure to new intellectual challenges may have fewer opportunities for doing so after leaving the workforce, which could otherwise impart satisfaction. Overall, personality may work in diverse ways to determine well-being when individuals leave the workforce in later life, and thus it is an empirical question to determine which effect is stronger.

## 3. Model and empirical strategy

Following Blanchflower and Oswald [5], we assume that well-being can be described by the following function

r=g(f(p,p∙n,x,t))+e (1)

where *r* is the level of well-being (satisfaction with life, income and leisure) reported in the survey. The *f*(·) function represents actual well-being which is known only to the individual asked in the survey; *g*(·) is a non-differentiable function linking actual to reported well-being; *p* is a vector of pathways of leaving one’s work; *n* is a vector of personality traits; *x* is a vector of individual characteristics; *t* is time; and *e* is the error term. Here our main hypothesis is that the well-being effect of exiting the labor market through alternative pathways may depend on the individual’s personality characteristics. The empirical counterpart of (1) can be written out as

$$W_{it}=a_{0}+a_{1}{Paths}_{it}+a_{2}{BigFive}_{it}+a_{3}({Paths}_{it}\times {BigFive}_{it})+a_{4}X_{it}+T_{t}+u_{i}+\varepsilon_{it}$$ (2)

where *W*_*it*_ represents satisfaction with life, income and leisure of individual *i* at time *t*, *Paths*_*it*_ is a vector of dummy variables indicating pathways of leaving work, *BigFive*_*it*_ is a vector of the Big Five personality traits, *X*_*it*_ is a vector of time-varying predictor variables, and *T*_*t*_ is a vector of time dummies which capture trends in well-being that are common to all individuals. The two additional terms *u*_*i*_ and *ε*_*it*_ are the error terms; *u*_*i*_ is the person-specific error (or individual fixed effects) and *ε*_*it*_ is the idiosyncratic error.

Estimating Eq (2) presents the challenge that the exit pathways might be endogenous. The practical difficulty here is finding a suitable instrument for each possible exit pathway. Lee and Kim [34] and Palomäki [11] recommend using a fixed effects model which allows to consider how within-person changes in employment status–from employment to being out of the labor force in different statuses–relate to within-person changes in well-being. Given that the fixed effects estimator focuses only on within-person variation, it helps relate changes in individuals’ employment status with their well-being, after controlling for unobserved heterogeneity [35].

Another empirical issue concerns our measures of personality. Previous studies have demonstrated that personality is subject to change. It changes at least as much as other factors that are often considered to be variable, including income and unemployment [36], and such changes occur throughout a person’s life course [37, 38]. In addition, within-person changes in personality are linked with within-person changes in well-being [36].

To reduce the potential endogeneity problem, Bowles et al. [15] and Boyce and Wood [25] recommend using *pre-event* personality; that is, personality scores that were collected before the respondent exited the labor market. We follow the same empirical strategy here. Accordingly, we limit our sample to include only those who were working in 2005. These individuals might have left their work in the subsequent time-points in 2006, 2007 and 2008. In that way, we can measure the impact of personality on overall life, income, and leisure satisfaction for all subsequent years. The pre-event personality traits measured in 2005 (the only year providing data on personality traits in the BHPS) are free from any potential influence of exiting the workforce. Thus Eq (2) can be rewritten as follows

$$W_{i,t=1,2,3}=a_{0}+a_{1}{Paths}_{i,t=1,2,3}+a_{2}{BigFive}_{i,t=0}+a_{3}({Paths}_{i,t=1,2,3}\times {BigFive}_{i,t=0})+a_{4}X_{i,t=1,2,3}+T_{t=1,2,3}+u_{i}+\varepsilon_{i,t=1,2,3}$$ (3)

where *t* = 0 denotes the year 2005, and *t* = 1,2,3 denotes the years 2006, 2007 and 2008, respectively.

Considering the model given by Eq (3), our parameters of interest are in the vector *a*_1_, on the pathways of leaving work, as well as in the vector *a*_3_, on the interaction terms between personality and exit pathways. As explained earlier, personality may change and thus would not be absorbed by the fixed effects in the model. Ideally, one would like to have personality measures available over several years, but this is not feasible in our case. In the BHPS, personality measures are observed only once. What this implies is that the main effect of personality corresponding to the vector *a*_2_ naturally drops out from the fixed effects estimation, but the interactions with the pathways remain [28]. Finding any or all of the coefficient estimates within the vectors *a*_1_ and *a*_3_ are statistically significant would provide evidence that the effect of leaving work on satisfaction with life, income and leisure varies along the distribution of the Big Five personality traits.

To aid the interpretation of our results, we standardized both our well-being variables and the personality variables to have a mean of 0 and a standard deviation of 1. All our estimations are carried out using linear fixed effects models with robust standard errors clustered at the individual level [39]. We also relaxed the assumption of a linear model and confirmed that qualitatively equivalent results can be obtained using a fixed effects ordered logit model.

## 4. Data

Our data source is the British Household Panel Survey (BHPS). The BHPS, which was collected during the period 1991 to 2008, is a longitudinal household survey representative of the population that resides in the United Kingdom. Our sample consists of individuals aged between 50 and 75 years old at the time of the interviews who were working in 2005 but might have left work during the subsequent years from 2006 to 2008. Participants completed every year socio-economic measures and measures related to satisfaction with different domains of their life, while personality measures were completed only in year 2005.

As discussed earlier, our main analysis will be based on data collected from 2005 to 2008, given the importance of using personality scores that were collected before the respondent exited employment. After excluding observations with missing answers for the questions required for our analysis, the final sample corresponded to an unbalanced panel of 7,392 observations and 2,024 individuals. Our study has received written exemption from ethics review (No. 270/62) by The Research Ethics Review Committee for Research Involving Human Research Participants, Group I, Chulalongkorn University. All procedures performed in studies involving human participants were in accordance with the ethical standards of the institutional and/or national research committee and with the 1964 Helsinki declaration and its later amendments or comparable ethical standards.

### 4.1 Satisfaction with life and other domains

Our dependent variables originate from three separate measures capturing satisfaction with life, income and the amount of leisure time, all of which have been used in different disciplines including in economics (e.g., [7, 40–42]). A person’s overall satisfaction depends on satisfaction with specific domains. According to Rojas [43], human life can be partitioned in many possible ways, and although such partitions do not necessarily have additive effects on life satisfaction, they meaningfully relate to the way people think about their life. Hence, they can be useful for the understanding of overall life satisfaction. For this study, we selected two domain satisfactions that well-being research has identified as relevant when individuals exit the labor market at an older age. As explained earlier, the lifecycle model predicts changes in leisure and income satisfaction as well as changes in satisfaction with overall life, which in turn depend on the exit route from the labor market. The importance of such changes in a person’s life motivates our focus on those key areas.

The life satisfaction question asks individuals to evaluate how satisfied they are with their life overall, with answers ranging from 1 (not satisfied at all) and 7 (completely satisfied). Because leaving the workforce is usually accompanied by large changes in income and leisure, we also use satisfaction with income and the amount of free time (leisure) as our dependent variables. Responses to these two additional measures were also reported on the same 7-point scale where 1 indicates “not satisfied at all” and 7 indicates “completely satisfied”.

### 4.2 Pathways of leaving work

We define whether an individual is working or not using the BHPS job status questionnaire. Our sample consists of respondents who were working in 2005 but might have left their work in the subsequent time-points in 2006, 2007 and 2008. An individual who is engaged either in paid work or self-employed is defined as “working”. We also define four pathways of leaving work: (i) early retirement, (ii) mandatory retirement, (iii) ill health or family care, and (iv) unemployment. Because our interest is on the different pathways of leaving work, we focus on respondents who did not intend to immediately return to the labor market. We thus removed those respondents who were looking for a job in the past four weeks.

The four pathways of leaving work are defined as a set of dummy variables as follows. The dummy variable for the pathway related to mandatory retirement is equal to one if the respondent left the labor force by mandatory retirement in the years 2006–2008 and zero otherwise. Similarly, the dummy variable representing early retirement is equal to one if the respondent became an early retiree in the years 2006–2008 and zero otherwise. The dummy variable for ill health or family care takes the value one if the respondent exited the labor force for reasons related to ill health or family care in the years 2006–2008 and zero otherwise. Finally, the unemployment dummy is equal to one if the respondent became unemployed in the years 2006–2008 and zero otherwise. Note that a person exits to mandatory or early retirement only once, but he/she may transition in and out of ill-health/family care or unemployment. In other words, while the former two are absorbing states, the latter two are not.

Out of all 7,392 observations used in our analysis (years 2005–2008), 90.2% are defined as working and 9.8% are defined as not working. Accordingly, 6,666 observations (2,023 unique individuals) correspond to working respondents. The rest of the observations, 726 in total (242 unique individuals), are from non-working respondents among which 24.7% reported early retirement, 58.56% mandatory retirement, 13.2% ill health and/or family care, and 3.6% unemployment.

### 4.3 Personality measures

Personality is measured using the Big Five traits, which have been used in many existing studies (e.g., [25, 28, 44, 45]). The Big Five were obtained in the year 2005 by using a 15-item inventory. Each dimension is captured by the answers to 3 questions, and answers are coded on a scale of 1 to 7, where 1 indicates “does not apply to me at all” and 7 indicates “applies to me perfectly”. Each trait’s total score ranges from 3 to 21. Previous research [46] shows that these brief scales of the Big Five inventory demonstrate both strong internal coherence and satisfactory reliability. Cronbach’s α reliabilities across the entire BHPS sample in 2005 were 0.53 (agreeableness), 0.52 (conscientiousness), 0.54 (extraversion), 0.68 (neuroticism) and 0.67 (openness). In addition, Gosling et al. [47] provide empirical evidence supporting the use of a concise inventory for assessing personality.

### 4.4 Other explanatory variables

Our analysis controls for the potentially confounding effects of socio-economic variables that may be correlated with the different pathways of leaving work and personality while, at the same time, influencing satisfaction with life, income and leisure. These include age, age squared, real household income (the base year is 2008), a binary variable indicating educational attainment (General Certificate of Education Advanced Level), whether the respondent is self-employed, has children living in the same household, has seen a doctor or visited a hospital as outpatient, has stayed in a hospital as inpatient, as well as a set of dummy variables for marital status, demographic areas, and survey waves. A variance inflation factor analysis indicated no potential multicollinearity among the explanatory variables used in the subjective well-being regressions. The choice of covariates is consistent with the well-being literature [25, 28, 42, 48, 49].

### 4.5 Summary statistics

Table 1 shows summary statistics for all variables in our analysis by labor market status. Individuals who retired early as well as those who hit mandatory retirement reported higher satisfaction with life and leisure compared to those who were working. Early retirees were most satisfied with their income. On the contrary, individuals who left the labor market for reasons related to ill health or family care as well as because they became unemployed reported lower levels of satisfaction with overall life and income than those who were working. Overall, there is evidence that the specific pathway of leaving work matters in terms of satisfaction with life and the domains of income and leisure.

**Table 1:** Summary statistics (non-standardized) by exit pathway from the labor force.

Variable Means	All	Working	Mandatory retirement	Early retirement	Ill health or family care	Unemployment
Mandatory retirement	0.057					
Early retirement	0.024					
Ill health or family care	0.013					
Unemployment	0.004					
Life satisfaction	5.350	5.336	5.614	5.732	4.594	4.692
Income satisfaction	4.782	4.798	4.781	5.056	3.448	3.731
Leisure satisfaction	4.834	4.726	5.981	6.073	4.927	4.885
Male	0.542	0.545	0.438	0.715	0.458	0.654
Age	58.009	57.518	65.181	59.402	57.615	58.346
Real household income (thousand pounds, 2008 base year)	42.083	43.455	27.017	35.048	28.746	34.082
Having children living in the household	0.093	0.099	0.014	0.039	0.083	0.077
Never married	0.049	0.049	0.047	0.034	0.115	0.038
Separated	0.034	0.032	0.071	0.034	0.010	0.000
Divorced	0.084	0.085	0.075	0.028	0.115	0.115
At least A-level	0.425	0.431	0.318	0.520	0.333	0.423
Outpatient	0.733	0.723	0.812	0.782	0.927	0.846
Inpatient	0.066	0.060	0.106	0.067	0.344	0.077
Agreeableness	16.408	16.382	16.704	16.480	16.875	15.808
Conscientiousness	16.534	16.539	16.221	16.447	17.573	17.269
Extraversion	13.125	13.101	13.412	13.201	13.135	14.038
Neuroticism	10.348	10.344	10.212	9.877	12.104	10.154
Openness	13.409	13.442	12.835	13.235	14.010	13.308
Number of observations (N)	7,392	6,666	425	179	96	26

Source: BHPS waves 15–18.

Early retirees and those separating from the labor force by unemployment were disproportionately male. Not surprisingly, mandatory retirees were older on average than members of the other groups. Early retirees and those separating by unemployment had higher incomes than the others not working, but lower than those working. Consistent with their reason for leaving the labor force, the use of inpatient and outpatient healthcare was highest for those who separated because of ill health/family care.

There was no clear pattern of personality traits related to working status or reason for separating from the workforce. Each group was highest or lowest in at least one personality characteristic. Working people were lowest in extraversion. Those who were out of the labor market because of mandatory retirement were lowest in conscientiousness and openness. Early retirees were lowest in neuroticism. Those not working because of ill health/family care had the most extreme personalities. They were highest in conscientiousness, agreeableness, openness, and neuroticism. The unemployed were highest in extraversion and lowest in agreeableness.

## 5. Results

Table 2 reports preliminary estimates that do not account for potential interaction effects between pathways of leaving work and personality traits. This provides a baseline for interpreting the importance of personality on satisfaction with life, income and leisure when leaving work.

**Table 2:** Exit pathways and well-being.

	Life satisfaction	Income satisfaction	Leisure satisfaction
Early retirement	0.169**	-0.0622	0.552***
	(0.0778)	(0.0713)	(0.0930)
Mandatory retirement	0.00938	-0.239***	0.445***
	(0.0587)	(0.0525)	(0.0631)
Ill health or family care	-0.377***	-0.536***	0.0668
	(0.135)	(0.126)	(0.141)
Unemployment	-0.517***	-0.424***	0.249
	(0.190)	(0.152)	(0.204)
Constant	1.861	3.132	-3.435
	(2.739)	(2.756)	(2.725)
N observations	7,392	7,392	7,392
N individuals	2,024	2,024	2,024

Note

*p<0.1

**p<0.05

***p<0.01. Control variables include age, age squared/100, real household income, marital status, educational attainment, having children in the household, being inpatient, being outpatient, time and demographic areas. Robust standard errors are in parentheses. Source: BHPS waves 15–18.

The results suggest that early retirement is positively associated with life satisfaction and satisfaction with time spent in leisure, while it has a negative, albeit statistically insignificant relationship with income satisfaction. We also find that mandatory retirement is associated with lower income satisfaction but higher leisure satisfaction. When individuals confront leaving their work for reasons related to ill health or family care, they report lower satisfaction with their overall life and income. Becoming unemployed at an older age also tends to decrease both life and income satisfaction.

These findings reject the hypothesis of ‘homogeneous’ behavior across distinct subgroups of individuals, suggesting that an individual’s specific pathway matters in terms of well-being when leaving the workforce in later life.

We now add personality traits to test whether personality moderates the link between pathways of leaving work and satisfaction with life, income and leisure. The results appear in Table 3. Consistent with our earlier findings in Table 2, the direct effects of the different pathways of leaving work continue in this specification. But different personality traits moderate the effects of pathways in various ways.

**Table 3:** Exit pathways, personality traits and well-being.

	Life satisfaction	Income satisfaction	Leisure satisfaction
Early retirement	0.181**	-0.0544	0.543***
	(0.0791)	(0.0675)	(0.0928)
Mandatory retirement	0.0536	-0.248***	0.498***
	(0.0584)	(0.0544)	(0.0665)
Ill health or family care	-0.375**	-0.633***	-0.0335
	(0.150)	(0.133)	(0.138)
Unemployment	-0.557***	-0.423***	0.231
	(0.178)	(0.147)	(0.278)
Early retirement × Agreeableness	-0.103	-0.0989	0.136
	(0.0984)	(0.0793)	(0.107)
Early retirement × Conscientiousness	0.0187	0.0183	-0.114
	(0.0935)	(0.0715)	(0.0964)
Early retirement × Extraversion	-0.188**	-0.226***	-0.156*
	(0.0792)	(0.0814)	(0.0807)
Early retirement × Neuroticism	0.0724	0.0196	-0.00280
	(0.0896)	(0.0750)	(0.0839)
Early retirement × Openness	0.0151	0.0833	-0.0810
	(0.0782)	(0.0744)	(0.0942)
Mandatory retirement × Agreeableness	-0.131**	-0.0659	-0.160**
	(0.0604)	(0.0636)	(0.0639)
Mandatory retirement × Conscientiousness	0.0588	0.0611	0.132**
	(0.0600)	(0.0572)	(0.0650)
Mandatory retirement × Extraversion	-0.0753	0.0537	-0.0738
	(0.0646)	(0.0515)	(0.0579)
Mandatory retirement × Neuroticism	0.0506	-0.00295	0.0651
	(0.0578)	(0.0553)	(0.0595)
Mandatory retirement × Openness	0.0840	-0.124**	0.0604
	(0.0557)	(0.0586)	(0.0558)
Ill health or family care × Agreeableness	-0.355	-0.157	-0.0154
	(0.216)	(0.151)	(0.171)
Ill health or family care × Conscientiousness	0.141	0.169	0.0885
	(0.202)	(0.148)	(0.175)
Ill health or family care × Extraversion	0.0294	0.0399	0.257**
	(0.134)	(0.113)	(0.124)
Ill health or family care × Neuroticism	0.0911	0.238*	0.259
	(0.141)	(0.125)	(0.157)
Ill health or family care × Openness	-0.0833	-0.108	-0.227
	(0.189)	(0.128)	(0.198)
Unemployment × Agreeableness	-0.149	0.171	-0.0886
	(0.141)	(0.143)	(0.229)
Unemployment × Conscientiousness	0.526***	0.302*	0.256
	(0.196)	(0.172)	(0.365)
Unemployment × Extraversion	-0.141	0.0151	-0.168
	(0.129)	(0.130)	(0.122)
Unemployment × Neuroticism	-0.0245	0.479***	-0.160
	(0.247)	(0.177)	(0.225)
Unemployment × Openness	0.164	-0.0183	0.299
	(0.184)	(0.125)	(0.188)
Constant	1.520	3.371	-3.624
	(2.775)	(2.787)	(2.760)
N observations	7,392	7,392	7,392
N individuals	2,024	2,024	2,024

Note: See Table 2.

Agreeableness plays a significant role on how mandatory retirement affects leisure satisfaction. It is useful to explain how this result can be interpreted precisely. Mandatory retirement is estimated to increase satisfaction with leisure time by 0.498 standard deviations for an individual with average levels of agreeableness. However, for an individual who is one standard deviation above the average in agreeableness the effect is 0.338 (= 0.498–0.160) standard deviations. This is a quite sizeable effect: it implies that mandatory retirement has a smaller positive impact by up to 32% (0.160/0.498) for highly agreeable individuals compared to typical individuals. This finding reflects the fact that individuals high in agreeableness suffer when losing social relationships from their work, and thus may find it more difficult to cope.

Conscientiousness also matters for mandatory retirement. Individuals high in conscientiousness who hit mandatory retirement are rewarded with 0.132 standard deviations increase in leisure satisfaction relative to those with average conscientiousness levels. Moreover, the life satisfaction of highly conscientious individuals is less affected from unemployment. The interaction term between becoming unemployed and conscientiousness attracts a positive and statistically significant coefficient. This effect is large and implies a 94% (= 0.526/0.557) difference between highly conscientious and typical individuals.

Consistent with our expectations, it turns out that highly conscientious individuals find it easier than typical individuals to cope when leaving the workforce; conscientiousness acts as a psychological buffer. This hypothesis was based on past research showing that conscientiousness is associated with the tendency to be proactive [50]. Thus, besides augmenting leisure satisfaction for those who hit mandatory retirement, conscientiousness augments life satisfaction for those becoming unemployed.

While agreeableness and conscientiousness seem to mainly affect individuals who hit mandatory retirement, extraversion appears to play a significant role for those who decide to retire early, an impact consistent across life domains. Perhaps most important, we find that extraverted individuals suffer a life satisfaction penalty of 0.188 standard deviations, a result suggesting a difference of about 104% (= 0.188/0.181) relative to typical individuals. This may be an indication that extraverted individuals who tend to be sociable, likeable, and outgoing may suffer when losing social relationships from their work, at least in the short run. Not surprisingly, we also find that the leisure satisfaction of extraverted individuals who exit the workforce for reasons related to ill health or family care is not affected in a negative manner; the interaction term between exiting the workforce due to ill health or family care and extraversion attracts a positive and statistically significant coefficient.

Table 3 further suggests that neuroticism matters for those who become unemployed. While typical individuals who become unemployed experience a decrease in income satisfaction of 0.423 standard deviations, for those high in neuroticism the negative effect is completely offset; that is, 0.479–0.423 = 0.056.

Previous research has documented that neuroticism relates to the tendency of appraising one’s life negatively [51]. We therefore expected to find that neuroticism would make it more difficult to cope when leaving the workforce. However, we were surprised to find that neuroticism augments income satisfaction for those who become unemployed. It may be that people high in neuroticism had a lower “baseline level” of income satisfaction relative to typical individuals so they were not affected as much. Yap et al. [26] provide a similar explanation for the role played by neuroticism in the context of other major life events including childbirth and widowhood.

Finally, individuals high in openness who hit mandatory retirement confront an income satisfaction penalty of 0.124 standard deviations; a difference of about 50% (= 0.124/0.248) compared to individuals with average openness levels. It may be that individuals high in openness who value exposure to new intellectual challenges have fewer opportunities for doing so after leaving the workforce, which could otherwise impart satisfaction.

Overall, our findings indicate that the well-being effects of exiting the labor force are shaped by three sources of individual heterogeneity: (i) the specific exit route, (ii) satisfaction with overall life and the domains of income and leisure, and (iii) the Big Five personality traits. Depending on the exit route and satisfaction with life domain, different personality traits act as moderators.

## 6. Robustness and other checks

In this section we consider a number of ways in which our analysis may be extended. To find a more nuanced understanding of how personality might interact with workforce separation, we divide our sample into two groups using the socioeconomic class of the job the individual had before exiting the labor force: (i) routine level of work, and (ii) intermediate and professional level of work. Table 4 displays the results.

**Table 4:** Exit pathways, personality traits and well-being by the last job type.

	Routine work			Intermediate and professional work		
	Life satisfaction	Income satisfaction	Leisure satisfaction	Life satisfaction	Income satisfaction	Leisure satisfaction
	(1)	(2)	(3)	(4)	(5)	(6)
Early retirement	0.0521	-0.0714	0.409**	0.267***	-0.0624	0.664***
	(0.148)	(0.129)	(0.187)	(0.0953)	(0.0729)	(0.105)
Mandatory retirement	0.0361	-0.392***	0.543***	0.0702	-0.188***	0.505***
	(0.107)	(0.103)	(0.111)	(0.0685)	(0.0636)	(0.0831)
Ill health or family care	-0.595***	-0.665***	-0.235	-0.259	-0.752***	-0.00708
	(0.216)	(0.191)	(0.171)	(0.193)	(0.200)	(0.200)
Unemployment	-0.613***	0.0463	0.436	-0.530**	-0.480***	0.269
	(0.144)	(0.288)	(0.305)	(0.269)	(0.134)	(0.399)
Early retirement × Agreeableness	-0.164	-0.134	0.245	-0.110	-0.129	0.115
	(0.195)	(0.134)	(0.223)	(0.118)	(0.0912)	(0.124)
Early retirement × Conscientiousness	0.0536	0.135	-0.135	0.0390	-0.00866	-0.190
	(0.127)	(0.124)	(0.159)	(0.130)	(0.0884)	(0.120)
Early retirement × Extraversion	-0.0473	-0.0105	-0.131	-0.270***	-0.284***	-0.169*
	(0.121)	(0.122)	(0.162)	(0.0867)	(0.0872)	(0.0899)
Early retirement × Neuroticism	-0.0559	-0.146	0.279	0.0911	0.0373	-0.116
	(0.197)	(0.195)	(0.174)	(0.105)	(0.0700)	(0.0981)
Early retirement × Openness	-0.158	0.0469	-0.120	0.149*	0.0965	-0.0540
	(0.160)	(0.151)	(0.168)	(0.0891)	(0.0763)	(0.101)
Mandatory retirement × Agreeableness	-0.188*	0.0381	-0.293***	-0.0737	-0.112*	-0.0636
	(0.100)	(0.125)	(0.106)	(0.0728)	(0.0676)	(0.0712)
Mandatory retirement × Conscientiousness	0.107	0.0591	0.180**	-0.0260	-0.00361	0.0786
	(0.104)	(0.116)	(0.0917)	(0.0701)	(0.0608)	(0.0962)
Mandatory retirement × Extraversion	-0.0269	0.0852	0.0330	-0.0765	-0.00755	-0.145*
	(0.106)	(0.0852)	(0.0902)	(0.0729)	(0.0541)	(0.0782)
Mandatory retirement × Neuroticism	0.205**	0.110	0.130	-0.0635	-0.114*	0.0368
	(0.0868)	(0.0814)	(0.0959)	(0.0702)	(0.0671)	(0.0804)
Mandatory retirement × Openness	0.148	-0.117	0.0630	-0.0126	-0.145*	0.0378
	(0.0927)	(0.0941)	(0.0741)	(0.0702)	(0.0771)	(0.0784)
Ill health or family care × Agreeableness	-0.784***	-0.592***	-0.153	-0.0205	0.216	0.256
	(0.272)	(0.229)	(0.260)	(0.263)	(0.192)	(0.228)
Ill health or family care × Conscientiousness	0.562**	0.384*	0.436	-0.0144	0.0884	-0.140
	(0.257)	(0.198)	(0.301)	(0.229)	(0.187)	(0.201)
Ill health or family care × Extraversion	0.393**	-0.0116	0.438**	-0.219	0.0340	0.205
	(0.179)	(0.204)	(0.185)	(0.171)	(0.148)	(0.157)
Ill health or family care × Neuroticism	0.307	0.266	0.483**	0.0807	0.314**	0.260
	(0.195)	(0.192)	(0.194)	(0.209)	(0.146)	(0.265)
Ill health or family care × Openness	-0.208	0.0615	-0.601***	0.0854	-0.0136	0.0861
	(0.145)	(0.219)	(0.167)	(0.290)	(0.173)	(0.315)
Unemployment × Agreeableness	-0.358***	0.274	0.0139	-0.276	-0.325**	-0.441
	(0.113)	(0.183)	(0.234)	(0.223)	(0.130)	(0.540)
Unemployment × Conscientiousness	0.637**	-0.421	0.490	0.674***	0.513***	0.141
	(0.290)	(0.350)	(0.315)	(0.223)	(0.127)	(0.493)
Unemployment × Extraversion	-0.150	-0.468***	-0.286*	0.354*	0.414***	-0.0906
	(0.141)	(0.151)	(0.166)	(0.195)	(0.0858)	(0.363)
Unemployment × Neuroticism	-0.695***	0.724***	0.0462	0.781**	0.745***	-0.518
	(0.131)	(0.201)	(0.218)	(0.338)	(0.142)	(0.664)
Unemployment × Openness	0.419***	0.635**	0.323	-0.270	-0.489***	0.249
	(0.127)	(0.249)	(0.237)	(0.244)	(0.101)	(0.461)
Constant	-1.312	4.513	-2.718	2.345	0.339	-6.164*
	(4.994)	(5.453)	(4.629)	(3.442)	(3.141)	(3.328)
N observations	2,797	2,797	2,797	4,595	4,595	4,595
N individuals	836	836	836	1,336	1,336	1,336

Note: See Table 2.

There is evidence of substantial heterogeneity by job type, which in turn is more pronounced for three out of four exit pathways: early retirement, mandatory retirement, and ill health/family care. In terms of early retirement, personality plays a significant role for those with intermediate or professional jobs but not routine jobs. Extraverted individuals with intermediate or professional jobs who decide to retire early suffer a satisfaction penalty, an impact consistent across life domains. Turning to those who hit mandatory retirement, personality matters only for routine jobs. Agreeableness mitigates leisure satisfaction, conscientiousness augments leisure satisfaction, and neuroticism augments life satisfaction.

Among those who confront leaving their work for reasons related to ill health or family care, personality plays a more prominent role for routine jobs, with all personality traits displaying statistically significant interaction terms. Agreeableness mitigates life and income satisfaction, conscientiousness augments life satisfaction, extraversion augments life and leisure satisfaction, neuroticism augments leisure satisfaction, and openness mitigates leisure satisfaction. For intermediate or professional jobs, however, only one interaction term attracts a significant coefficient: neuroticism augments income satisfaction.

Overall, there is evidence of substantial heterogeneity according to the individual’s job type before exiting the labor force. Personality impacts more those with routine jobs if they hit mandatory retirement or exit the labor market because of ill health/family care. Meanwhile, personality impacts more those with intermediate or professional jobs if they retire early.

To address the concern that people with certain personality characteristics might select themselves to leave their work, we estimate a random effects logit model in which the dependent variable is the probability of leaving work through any of the four possible pathways (early retirement, mandatory retirement, ill health or family care, and unemployment). Table 5 displays the results: we first estimate a baseline specification with personality traits as the only predictor variables in column 1, and then add the other controls in column 2.

**Table 5:** Random effects logit estimates of the Big Five personality traits on the probability of leaving work.

	Without controls	With controls
Agreeableness	0.0712**	0.0421
	(0.0328)	(0.0511)
Conscientiousness	-0.0319	0.0623
	(0.0316)	(0.0485)
Extraversion	0.0479*	0.0645
	(0.0259)	(0.0399)
Neuroticism	0.0103	0.0346
	(0.0224)	(0.0362)
Openness	-0.0511**	-0.0268
	(0.0244)	(0.0380)
Constant	-4.638***	-83.37***
	(0.727)	(15.05)
N observations	7,392	7,392
N individuals	2,024	2,024

Note

*p<0.1

**p<0.05

***p<0.01. Control variables in column 2 include age, age squared/100, household size, real household income, marital status, educational attainment, having children in the household, being inpatient, being outpatient, time and demographic areas. Source: BHPS waves 15–18.

Without additional controls, we find that the estimated coefficient on agreeableness and openness is significant at the 5% level. When the other predictors in our main analysis are included, however, we do not find any evidence that personality influences the probability of leaving work. This implies that we can safely rule out possible selection effects–that a person might select to leave the labor force because of his/her personality traits.

Our analysis relies on a fixed effects model which controls for the potentially confounding effects of time-invariant unobserved heterogeneity. However, fixed effects do not control for everything. Consider early retirement, for example, which is often a choice and thus is likely to be an endogenous variable. If that is the case, then the inclusion of early retirees might also bias the estimated coefficients of the other control variables included in our model. Although following Terza et al. [52] we cannot reject the null hypothesis that early retirement is an exogenous variable, as a robustness check, we re-conducted the analysis excluding those individuals who retired early. As is evident from Table 6, our results remain unchanged, thus lending support for our earlier empirical strategy.

**Table 6:** Exit pathways, personality traits and well-being excluding those who retired early.

	Life satisfaction	Income satisfaction	Leisure satisfaction
Mandatory retirement	0.0549	-0.229***	0.491***
	(0.0588)	(0.0544)	(0.0676)
Ill health or family care	-0.441***	-0.619***	-0.0512
	(0.164)	(0.131)	(0.138)
Unemployment	-0.578***	-0.415***	0.239
	(0.178)	(0.143)	(0.270)
Mandatory retirement × Agreeableness	-0.119*	-0.0567	-0.172***
	(0.0617)	(0.0638)	(0.0666)
Mandatory retirement × Conscientiousness	0.0492	0.0459	0.137**
	(0.0624)	(0.0580)	(0.0680)
Mandatory retirement × Extraversion	-0.0723	0.0501	-0.0680
	(0.0647)	(0.0504)	(0.0589)
Mandatory retirement × Neuroticism	0.0595	-0.0174	0.0794
	(0.0579)	(0.0551)	(0.0607)
Mandatory retirement × Openness	0.0814	-0.125**	0.0591
	(0.0562)	(0.0585)	(0.0567)
Ill health or family care × Agreeableness	-0.336	-0.187	-0.0148
	(0.238)	(0.150)	(0.174)
Ill health or family care × Conscientiousness	0.214	0.163	0.0939
	(0.238)	(0.149)	(0.184)
Ill health or family care × Extraversion	0.0272	0.0114	0.281**
	(0.140)	(0.112)	(0.133)
Ill health or family care × Neuroticism	0.178	0.257**	0.272*
	(0.163)	(0.125)	(0.159)
Ill health or family care × Openness	-0.0572	-0.0645	-0.248
	(0.197)	(0.126)	(0.207)
Unemployment × Agreeableness	-0.0971	0.167	-0.0562
	(0.145)	(0.139)	(0.235)
Unemployment × Conscientiousness	0.507**	0.300*	0.182
	(0.198)	(0.165)	(0.327)
Unemployment × Extraversion	-0.106	0.0891	-0.186
	(0.120)	(0.114)	(0.130)
Unemployment × Neuroticism	0.0221	0.554***	-0.189
	(0.230)	(0.161)	(0.236)
Unemployment × Openness	0.161	-0.0320	0.299
	(0.177)	(0.113)	(0.207)
Constant	1.465	2.219	-3.187
	(2.784)	(2.788)	(2.789)
N observations	7,238	7,267	7,254
N individuals	2,025	2,026	2,025

Note: See Table 2.

We end this subsection by asking if the identified interaction effects between exit pathways and personality traits reflect heterogeneity between people in different labor market statuses. It could be argued, for example, that those who are unemployed differ substantially from those in other statuses, especially retirees. To shed some light on this issue, we re-conducted the analysis by dropping all unemployed observations. The estimates in Table 7 suggest that the results for this sub-sample are similar to our previous findings.

**Table 7:** Exit pathways, personality traits and well-being excluding those who are unemployed.

	Life satisfaction	Income satisfaction	Leisure satisfaction
Early retirement	0.175**	-0.0396	0.541***
	(0.0778)	(0.0672)	(0.0915)
Mandatory retirement	0.0567	-0.241***	0.488***
	(0.0584)	(0.0543)	(0.0667)
Ill health or family care	-0.427***	-0.629***	-0.0632
	(0.164)	(0.130)	(0.134)
Early retirement × Agreeableness	-0.0923	-0.0812	0.140
	(0.0968)	(0.0782)	(0.106)
Early retirement × Conscientiousness	0.00975	-0.0254	-0.120
	(0.0927)	(0.0776)	(0.0956)
Early retirement × Extraversion	-0.215***	-0.233***	-0.166**
	(0.0788)	(0.0792)	(0.0820)
Early retirement × Neuroticism	0.0706	0.0271	-0.00240
	(0.0864)	(0.0741)	(0.0816)
Early retirement × Openness	0.00356	0.0913	-0.0810
	(0.0777)	(0.0751)	(0.0949)
Mandatory retirement × Agreeableness	-0.130**	-0.0554	-0.159**
	(0.0609)	(0.0637)	(0.0644)
Mandatory retirement × Conscientiousness	-0.0726	0.0522	-0.0761
	(0.0644)	(0.0501)	(0.0576)
Mandatory retirement × Extraversion	0.0465	-0.0142	0.0680
	(0.0580)	(0.0545)	(0.0596)
Mandatory retirement × Neuroticism	0.0801	-0.122**	0.0606
	(0.0559)	(0.0582)	(0.0558)
Mandatory retirement × Openness	0.0575	0.0514	0.134**
	(0.0604)	(0.0572)	(0.0655)
Ill health or family care × Agreeableness	-0.281	-0.152	-0.0102
	(0.223)	(0.148)	(0.168)
Ill health or family care × Conscientiousness	0.117	0.144	0.127
	(0.204)	(0.145)	(0.176)
Ill health or family care × Extraversion	0.0355	0.0329	0.250**
	(0.133)	(0.115)	(0.125)
Ill health or family care × Neuroticism	0.128	0.251**	0.279*
	(0.148)	(0.123)	(0.157)
Ill health or family care × Openness	-0.0740	-0.0967	-0.242
	(0.192)	(0.128)	(0.198)
Constant	1.611	3.222	-3.166
	(2.762)	(2.777)	(2.763)
N observations	7,395	7,423	7,410
N individuals	2,025	2,025	2,025

Note: See Table 2.

## 7. Concluding remarks

In this paper we examine how different exit routes from the labor market are associated with individual well-being, and whether personality traits play a significant role by providing psychological insurance. Most of the earlier studies do not consider different exit pathways, and those who do abstain from analyzing the potential role of people’s personality traits as a psychological buffer.

Our findings provide a new perspective regarding the importance of personality on satisfaction with overall life and the domains of income and leisure when older individuals exit the labor market through alternative pathways. Analysis reveals that the well-being effects of exiting the labor force are shaped by three sources of individual heterogeneity: (i) the specific exit route, (ii) satisfaction with life, income and leisure, and (iii) the Big Five personality traits. Depending on the exit route and satisfaction with life domain, different personality traits act as moderators.

We find that perhaps the most important personality trait that acts as a psychological buffer is conscientiousness. Besides augmenting leisure satisfaction for those who hit mandatory retirement, conscientiousness augments life satisfaction for those becoming unemployed. On the contrary, extraversion mitigates satisfaction across all domains–life, income, and leisure–for those who retire early; an indication that extraverted individuals may suffer when losing relationships from their workplace. At the same time, extraversion may be helpful in augmenting leisure satisfaction for those who stop working for reasons related to ill health or family care. Neuroticism augments income satisfaction for those who become unemployed. This may reflect the fact that people high in neuroticism had a lower “baseline level” of income satisfaction so they were not affected as much. Finally, agreeableness mitigates life and leisure satisfaction for those hitting mandatory retirement, as is also the case with openness in terms of income satisfaction.

The current study is not without shortcomings. One limitation relates to the personality measures used in our analysis. Although the identification strategy we employed relies on pre-event personality to mitigate potential influences from exiting the workforce, personality is not randomized across the sample, meaning that any unobserved time-changing factors may still bias the estimates. Another issue are expectations: the perspective of leaving the labor market at time *t* may affect well-being at time *t*+1 and change personality. After all, people’s emotions are an inherent part of personality [53, 54].

In terms of the potential endogeneity of the different exit pathways, we performed robustness checks to mitigate this concern, but our results might still be confounded by reverse causality, for instance. It is possible that people with low levels of well-being self-select to exit the labor market through alternative routes compared to their counterparts with higher well-being. If that is the case, then we would be able to detect only a lower bound of the effects of exit pathways on well-being, meaning that our results would suffer from attenuation bias. Future research would benefit from different approaches attempting to address this issue, though we believe that correcting for reverse causality would leave our findings unchanged in a qualitative sense, but may reveal stronger effects. It is also worth noting that our results capture short-term changes in well-being, right after exiting the labor market. Zhu and He [4] and Kesavayuth et al. [55] investigate the well-being profile in the years leading up to and following retirement. Extending these studies to consider the potential role of exit routes and/or personality traits is a promising avenue for future research.

Finally, it is useful to discuss what the findings might tell us about public policy. Our findings shed some light on how personality works in terms of satisfaction with life and the domains of income and leisure when individuals leave the workforce in later life. As such they reveal which individuals may find it easier or more difficult to cope during this major life course transition. Knowing which individuals may experience reduced well-being when they stop working is a key element of optimal policy design.

We found that individuals scoring high on agreeableness, extraversion and/or openness who leave the workforce due to early or mandatory retirement might be particularly at risk. Policies and intervention programs should therefore focus on helping those individuals. While we do not suggest that people should receive public support based on their personality, intervention programs could be designed with the aim to raise awareness about the possible adverse effects on a person’s well-being of certain traits when separating from the labor force. Besides raising awareness, intervention programs could also encourage individuals to seek counselling, which can help alleviate troubling symptoms associated with lower satisfaction, so that people can adjust and function better as they transition out of the labor market. Given that population aging is a prevalent phenomenon, the study of the well-being effects of leaving work will likely continue to be an area of increased interest for academic research.
